# Nanoemulsion formulation of lemongrass essential oil using *Pseudomonas*-derived rhamnolipids for targeted phytopathogen suppression

**DOI:** 10.1007/s13205-025-04579-w

**Published:** 2025-10-28

**Authors:** Manemegalai Suria Gandi, Khairulmazmi Ahmad, Noriznan Mokhtar, Mohd Rafein Zakaria

**Affiliations:** 1 Institute of Plantation Studies, Universiti Putra Malaysia, Serdang, Selangor Malaysia; 2 Department of Bioprocess Technology, Faculty of Biotechnology and Biomolecular Sciences, Universiti Putra Malaysia, Serdang, Selangor Malaysia; 3 Department of Plant Protection, Faculty of Agriculture, University Putra Malaysia, Serdang, Selangor Malaysia; 4 Department of Process and Food Engineering, Universiti Putra Malaysia, Serdang, Selangor Malaysia

**Keywords:** Antifungal, Essential oil, Nanoemulsion, Rhamnolipid, Ultrasonication

## Abstract

The present study focuses on developing a bio-based oil-in-water (O/W) nanoemulsion formulation for pesticide application. Lemongrass (*Cymbopogon flexuosus*) essential oil (EO) was incorporated as the active agent, and rhamnolipid (RL) biosurfactant served as the emulsifier. RL exhibited more than 50% emulsification activity in all tested hydrophobic substrates and, at 1 g/L, it demonstrated a lower surface tension value (30.15 mN/m) than its synthetic counterpart, sodium dodecyl sulfate (SDS) (32.87 mN/m). Lemongrass EO exhibited a minimum inhibitory concentration MIC of 3.2 mg/mL against *Rigidoporus microporus* and *Fusarium oxysporum*, and 4.0 mg/mL against *Ganoderma boninense*, with minimum fungicidal concentration (MFC) ranging from 3.2 to 4 mg/mL. Nanoemulsions stabilized with RL were prepared at varying oil-to-surfactant ratios (OSR) using ultrasonication. The optimized formulation (A2) was prepared at an OSR of 1:2, corresponding to 10% oil (v/v) of the total emulsion volume. Formulation A2 exhibited a mean particle size of 119.95 nm with a polydispersity index (PDI) of 0.35. The formulation also showed favourable physicochemical properties, including a zeta potential of − 27.63 mV, viscosity of 22.56 mPa/s, and surface tension of 23.9 mN/m, along with good storage stability. The sonication time was optimized to 10 min to achieve minimal droplet size and PDI. Overall, this study demonstrates that RL-stabilized lemongrass EO nanoemulsions can serve as an eco-friendly nanofungicide, offering a sustainable alternative to synthetic fungicides in managing fungal phytopathogens.

## Introduction

Microbial diseases (caused by bacteria, fungi, viruses, and protozoa) in plants account for about 16% of agricultural yield losses, with fungal pathogens responsible for 70–80% of the total microbial infections (Murugan and Abd‐Elsalam, 2022). Unlike prokaryotic bacterial pathogens, fungal pathogens are eukaryotic organisms with more complex cellular structures, making their detection and management more challenging. Consequently, effective control of fungal infections requires comprehensive understanding of pathogen biology and innovative control strategies (Nazzaro et al. 2017). Recent advances in agricultural research have emphasised on the development of bio-based compounds to control plant diseases while reducing reliance on synthetic pesticides. Natural compounds, particularly plant-derived EOs, are highly favourable antimicrobial agents in agricultural systems due to their environmental compatibility, low toxicity, biodegradability, and high efficacy (Khetabi et al. 2022).

Plant EOs are secondary metabolites produced by aromatic plants (Felicia et al. 2022). They are rich in bioactive constituents such as limonene, citral, camphor, and carvacrol, which exhibit antifungal activity through multiple mechanisms (Fincheira et al. 2023). Their antifungal properties are largely attributed to lipophilic and low molecular weight terpenes and terpenoids, which disrupt fungal cell membranes, inhibit sporulation, and ultimately induce cell necrosis (Nazzaro et al. 2017). Lemongrass is a member of the Poaceae family native to India. Lemongrass EO is one of the commonly studied essential oils owing to its insecticidal, antimicrobial, and cytotoxic effects (Kumar et al. 2023). It contains citral, which is a mixture of two geometric isomers, neral and geranial, as the major chemical constituent, along with other important components such as geranyl acetate, geraniol, and citronellal (Bhatnagar 2020). However, the practical application of plant EOs is limited by their volatility and susceptibility to environmental degradation.

Nanoencapsulation of EOs has emerged as an effective strategy to overcome these constraints by enhancing EO stability and bioactivity. Nanotechnology has been widely employed in the pharmaceutical, cosmetics, food processing, and environmental sectors. In agriculture, nanoemulsion-based systems are increasingly explored as carriers for fertilizers, pesticides, and agrochemicals (Gupta et al. 2024). Nanoemulsions are colloidal dispersions of immiscible liquids with droplet sizes typically below 200 nm (Lakyat et al. 2024). They consist of a dispersed phase containing the bioactive compounds (i.e., flavor oils, EOs, and triglycerides) and a continuous phase (i.e., dimethyl sulfoxide (DMSO) or ultrapure water), stabilized by surfactants that reduce interfacial tension and prevent droplet coalescence (Barradas and De Holanda E Silva, 2020). Conventional nanoemulsions often employ synthetic surfactants such as Tweens and Spans (Mushtaq et al. 2023). Although effective, these synthetic surfactants are not ecofriendly and may negatively impact non-target organisms, ecosystems, and food safety (Arora et al. 2022).

Biosurfactants offer a more sustainable alternative as they are naturally produced by plants, animals, or predominantly by microorganisms through fermentation (Ali et al. 2024). Biosurfactants are classified into low- and high-molecular-weight groups based on their molecular mass, with glycolipids being the most extensively investigated low-molecular-weight biosurfactants (Kashif et al. 2022). RLs are a class of glycolipid biosurfactants produced primarily by *Pseudomonas aeruginosa*. (Sorour et al. 2025). They consist of di-L-rhamnose units linked to hydroxy fatty acid chains (Eslami et al. 2020). RLs have been applied in diverse sectors, including cleaning, food processing, cosmetics, and bioremediation (Kleinen 2023). More recently, RLs have gained attention in agriculture due to their antimicrobial properties. They have been utilized as biopesticides, antimicrobial agents, and compost additives (Guzmán et al. 2024). Their amphiphilic structure promotes strong emulsification, while their antifungal activity enhances their value in sustainable plant disease management (Rocha et al. 2020). Nanoemulsion-based treatments in agriculture are advantageous due to their high bioavailability, improved solubility, controlled release, and targeted delivery of active compounds (Gupta et al. 2024; Xiong et al. 2025). Incorporating biosurfactants such as RLs into EO-based nanoemulsions not only enhances emulsion stability but also adds intrinsic antimicrobial properties. Hence, this study aimed to develop and characterize a lemongrass EO-loaded nanoemulsion stabilized by RLs and to evaluate its antifungal activity against fungal phytopathogens.

## Materials and methods

### Materials

The lemongrass EO was purchased from Evoke Occu, Malaysia. The RL was produced via batch fermentation in a 1.5 L Minifors 2 bench-top bioreactor (Infors HT, Switzerland) equipped with two Rushton turbine impellers, an optical dissolved oxygen (DO) sensor (VisiFerm, Hamilton, USA), and an analog pH sensor (Easyferm Plus Arc, Hamilton, USA). Biodiesel side stream waste glycerol, obtained from the Biomass and Biorefinery Laboratory, Universiti Putra Malaysia, Selangor, Malaysia, was used as the substrate and pretreated according to the method described by Baskaran et al. (2021). *P. aeruginosa* RS6 was procured from the Institute of Bioscience, Universiti Putra Malaysia, and maintained as a glycerol stock at − 80 °C. The strain was originally isolated from Sri Serdang Lake, Selangor, Malaysia (Nordin et al. 2013).

### Structural and physicochemical characterization of RL

#### Emulsification activity of RL

The emulsification activity of RLs was assessed to determine the ability of RLs to form emulsions. Fresh fermentation broth was autoclaved, cooled to room temperature and, subsequently centrifuged at 14,758 × g for 10 min at 4 °C. In this test, 2 mL of cell-free RL supernatant was added to 2 mL of hydrophobic substrates (cooking oil, diesel, kerosene, and crude petroleum) in test tubes and vortexed for 2 min using a vortex mixer (IKA, Staufen, Germany). Then, the mixtures were left at room temperature (25 ± 2 °C) without further agitation. SDS served as the positive control, whereas distilled water was used as the negative control. The emulsification index was recorded after 1(E_24_) and 30 (E_720_) days using the formula below (Sharma et al. 2017):

1 $${\text{Emulsification}}\,{\text{index}} \left( \% \right) = \frac{{{\text{the}}\,{\text{height}}\,{\text{of}}\,{\text{the}}\,{\text{emulsion}}\,{\text{layer}}}}{{{\text{total}}\,{\text{height}}\,{\text{of}}\,{\text{the}}\,{\text{mixture}} }} \times 100$$

#### Surface tension of RL

Surface tension values of RLs at different concentrations were determined using an automated surface tensiometer (Kibron Inc., Finland) according to the Du Nouy ring method (Baskaran et al. 2021). In this analysis, a needle-like probe was used in place of the traditional ring. RL solutions were placed in dyne cups on the measurement tray. Before each measurement, the tensiometer was calibrated using distilled water, and the probe was flame-sterilized. All measurements were conducted at 25 ± 1 °C. During measurement, the dyne cup was raised until it made contact with the probe, and readings were recorded once equilibrium was achieved. For each concentration, the mean surface tension was calculated from three independent measurements.

### Fourier transform infrared spectroscopy (FT-IR) of rhamnolipid

The functional groups and chemical bonds of the purified RLs were characterized using FTIR spectroscopy (Agilent Technologies, USA). Spectra were acquired over the range of 4000–650 cm⁻^1^ at a resolution of 4 cm⁻^1^ with four scans per sample. A standard purified RL (Agae Technologies, USA) was included as a reference.

### Antifungal activity of lemongrass EO

The antagonistic activity of lemongrass EOs was evaluated against *G. boninense*, *R. microporus,* and *F.oxysporum,* using agar well diffusion assay following the method described by Dangol et al. (2023) with minor modifications. The test concentrations of lemongrass EO were 0.8, 1.6, 2.4, 3.2, and 4.0 mg/mL. Agar plates were prepared by adding a specific volume of lemongrass EO into cooled molten PDA (45 °C), followed by swift manual rotation in a sterile conical flask to ensure miscibility. Subsequently, the mixture was poured into sterile Petri plates and allowed to solidify. Upon solidification, a 6-mm mycelial disc was removed from the edge of a 7-day-old fungal culture using a sterile cork borer and inoculated at the center of the treated Petri plates. Untreated Petri plates act as the negative control. Benomyl 50WP was used as the positive control. To prevent evaporation of EOs and any possible contamination, the plates were sealed with laboratory parafilm. All plates were incubated at 25 ± 2 °C. Each treatment and control was performed in triplicate. Data was recorded once the negative control plates exhibited full mycelial growth. The percentage of mycelial inhibition was calculated using the formula below (Devi et al. 2021):

2 $${\text{Percentage}}\,{\text{of}}\,{\text{mycelial}}\,{\text{inhibition}}\,{\text{growth}} \left( \% \right) = \frac{C - T}{C} \times 100$$

where C = Growth of fungus in control and T = Growth of fungus in treatment.

To determine the fungicidal effects of lemongrass EO, a 6-mm PDA plug was reinoculated from plates showing no growth or suppressed growth onto unamended PDA medium. After an additional seven days of incubation at 25 ± 2 °C, the plates that did not show any mycelial growth were considered fungicidal against the test pathogens (Devi et al. 2021).

### Preparation of nanoemulsions

Lemongrass EO, RL, and ultrapure water were used to prepare nanoemulsions. The formulations were prepared using a high-energy emulsification approach, ultrasonication. The formulations were prepared at the different OSRs as listed in Table 1. The oil composition was fixed for all formulations. Firstly, the dispersed phase was prepared by mixing the oil and surfactant on a hotplate stirrer at 250 rpm at ambient temperature. Ultrapure water (aqueous phase) was then added dropwise to the dispersed phase while stirring. Upon forming the coarse emulsions, the samples were subjected to ultrasonication. The emulsions were sonicated for 3 min at 40% amplitude with 30 s on/30 s off pulses. To minimize overheating, the samples were placed in an ice bath.

**Table 1:** Composition of nanoemulsions developed in this study

Composition	*C. flexuosus* essential oil (v/v %)	Rhamnolipid (v/v %)	Ultrapure water (v/v %)
A1	10	10	80
A2	10	20	70
A3	10	30	60

### Stability tests of nanoemulsions

The developed nanoemulsions were subjected to centrifugal, thermal, and storage stability tests. For centrifugal stability, all the developed nanoformulations were centrifuged at 1160 × *g* for 15 min and stored for four weeks at room temperature (28 °C) and observed for signs of destabilization. For thermal stability, the nanoformulations were stored at 54 °C for two weeks and at 4 °C for one week, after which the formulations were visually inspected for phase separation. For storage stability, the nanoformulations were stored at ambient temperature for 30 days, and the droplet size and PDI were measured on days 0, 7, 14, 21, and 28 (Ahmad et al. 2015).

### Physicochemical characterization of nanoemulsions

All the formulated nanoemulsions were evaluated for their particle size and PDI, zeta potential, pH, and viscosity. The sonication time of the selected nanoemulsion formulation was optimized.

### Particle size and PDI analysis

The droplet size and PDI measurements of the nanoemulsions were performed by dynamic light scattering using a zetasizer (Zetasizer NanoPlus, Particulate Systems, UK) as described by Mohammed et al. (2020). Undiluted samples were transferred into cuvettes and inserted into the equipment’s cell.

### Zeta potential evaluation

Zeta potential indicates the surface charge and reflects the colloidal stability of emulsion systems. The zeta potential values of the developed nanoemulsions were determined using a zetasizer (Zetasizer NanoPlus, Particulate Systems, UK) (Sun et al. 2023). Clean capillary cells were flushed with distilled water before sample introduction. Undiluted samples were introduced directly into the cell, and measurements were taken.

### pH

The pH of the samples was measured using a Delta 320 pH meter (Mettler-Toledo, Switzerland) by placing the electrode directly into the samples. The pH meter was calibrated using standard buffers of pH 4, 7, and 9 before use. All measurements were taken at room temperature (25 ± 2 °C).

### Viscosity evaluation

The dynamic viscosity of the developed nanoemulsions was measured using a dynamic viscometer (Rheolab QC, Anton Paar, USA) following the method described by Teixeira et al. (2017). Approximately 20 mL of sample was placed in a viscometer’s cup, and the device was allowed to start running. The rotational speed (rpm) of the viscometer was carefully adjusted to ensure accuracy of the viscosity values obtained.

### Optimization of nanoemulsion

Sonication time of the selected nanoemulsion was optimized by subjecting the nanoformulation to ultrasonication for 5, 10, and 15 min at 40% amplitude with a 30 s on/30 s off pulse.

### Statistical analysis

All treatments and controls were conducted in triplicate. The data obtained are expressed in mean ± standard deviation. Quantitative results were analysed using analysis of variance (ANOVA) in SPSS software (version 23). Tukey’s HSD test at a 5% level of significance (*p* = 0.05) was used to compare the mean values of different treatments in the experiments.

## Results and discussion

### Emulsification activity of RL

**Fig. 1:**
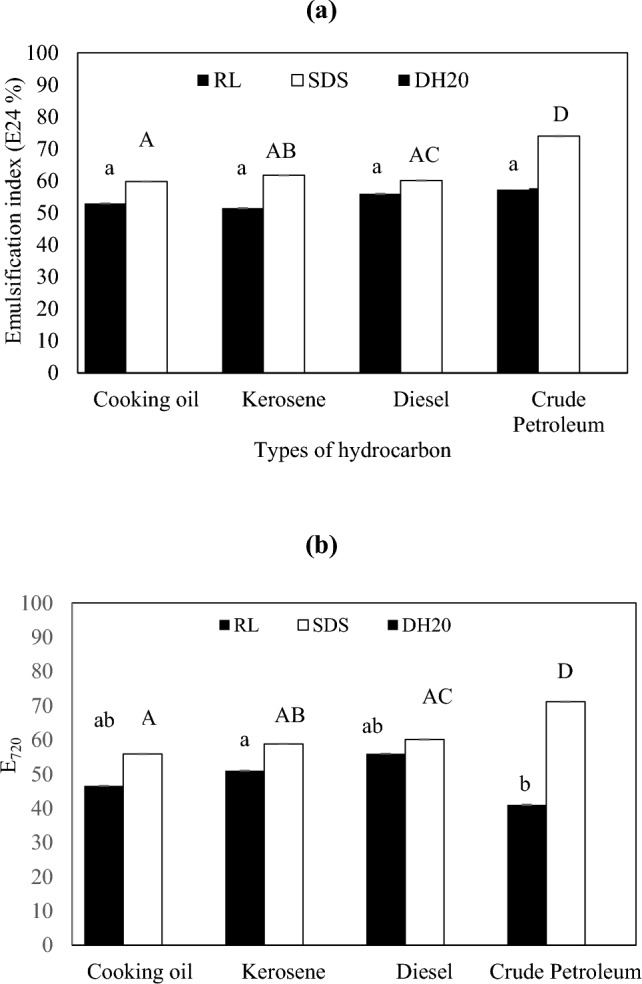
Emulsification activity of RLs, SDS and distilled water against different hydrophobic substrates after (**a**) 1 day and (**b**) 30 days of incubation at room temperature (24 °C)

The stability of RL-stabilized emulsions was evaluated by measuring the emulsification index after 1and 30 days. After 1 day, RL and SDS reported the highest E_24_ against crude petroleum at 57.69% and 73.97%, respectively (Fig. 1a). The lowest E_24_ was reported by RL on kerosene (51.47%), while SDS reported the lowest E_24_ on cooking oil (59.80%). Nevertheless, RL recorded an E_24_ of above 50% on all hydrophobic substrates, while SDS showed E_24_ above 60% on all hydrophobic media except cooking oil. The emulsification activity recorded by SDS was comparatively higher than RL on all the substrates. Distilled water did not show emulsification activity on any of the substrates. The difference in the emulsification index may be attributed to the chain size of the hydrophobic sources (Lovaglio et al. 2011). Cooking oil, for instance, is composed of a complex mixture of fatty acids, while kerosene is made up of long-chain hydrocarbons. Pornsunthorntawee et al. (2008) reported that the emulsifying activity of RL in crude oil and vegetable oil was higher than in short-chain substrates, which was evident in this study.

After 30 days, the E_720_ of RL remained stable in all media except for crude petroleum (Fig. 1b). The E_720_ of diesel (55.95%) and kerosene (50.98%) remained similar to values reported on day 1. Cooking oil, however, reported a slight decrease in E_720_ (46.57%). This could be due to gradual droplet coalescence and minor rearrangement of free fatty acids and triglycerides at the interface, leading to a modest decline in emulsion stability during storage (Xiao et al. 2025). Crude petroleum showed a significant reduction in E_720_ by dropping to 41.03%. This may be attributed to the complex composition and high viscosity of the long-chain hydrocarbon, which caused desorption of rhamnolipids at the oil–water interface, promoting droplet coalescence (Al-Sakkaf and Onaizi 2023a, b). Meanwhile, the E_720_ of SDS on all the hydrophobic substrates remained similar, with a slight reduction in certain media (~ 4%).

Due to microbial fermentation and subsequent downstream processing, biosurfactants like RLs are known to have higher production and purification costs than traditional synthetic surfactants like SDS (Kumari et al. 2023). Nevertheless, RLs offer several advantages that may offset their higher initial cost. This includes biodegradability, a lower environmental impact, and inherent antimicrobial activity that could improve the efficacy and reduce the need for additional active ingredients (Kabeil et al. 2025). Moreover, ongoing developments in large-scale fermentation technology and the utilization of inexpensive substrates are expected to gradually narrow the price gap between biosurfactants and synthetic surfactants, thereby enhancing the economic feasibility of RLs in agricultural applications (Paul et al. 2025).

### Surface tension of RL

**Fig. 2:**
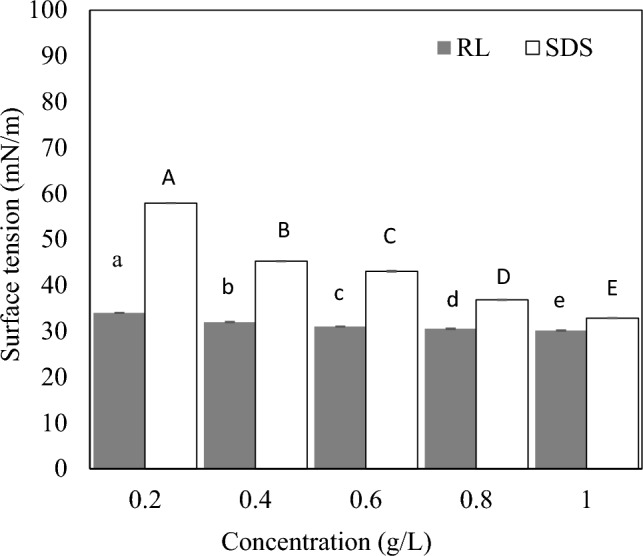
Surface tension of RLs and SDS at different concentrations

Surfactants play a pivotal role in reducing the surface tension of liquids, thereby stabilizing interfacial systems. In this study, the ability of RL and SDS to reduce surface tension of water at different concentrations was evaluated (Fig. 2). Across all tested concentrations, the cell-free supernatant containing RLs exhibited higher surface tension reduction than SDS. At the lowest tested concentration (0.2 g/L), RL reported a surface tension of 34 mN/m, whereas SDS exhibited 57.94 mN/m. The surface tension gradually decreased as the concentration of the tested surfactants (RL and SDS) increased. At 1 g/L, RL showed a surface tension of 30.15 mN/m, while SDS showed 32.87 mN/m. This finding indicates that RLs possess greater surface tension reduction capacity than SDS. The present finding is in line with Mendes et al. (2015), who reported a surface tension of approximately 27 mN/m for RLs at 150 mg/L and 36 mN/m for SDS. Additionally, El-Housseiny et al. (2020) demonstrated that both cell-free supernatant and aqueous RL solution effectively lowered water surface tension from 72 mN/m to 36 mN/m. This shows that RLs of varying levels of purity are able to effectively reduce the surface tension of water.

### FT-IR of RL

**Fig. 3:**
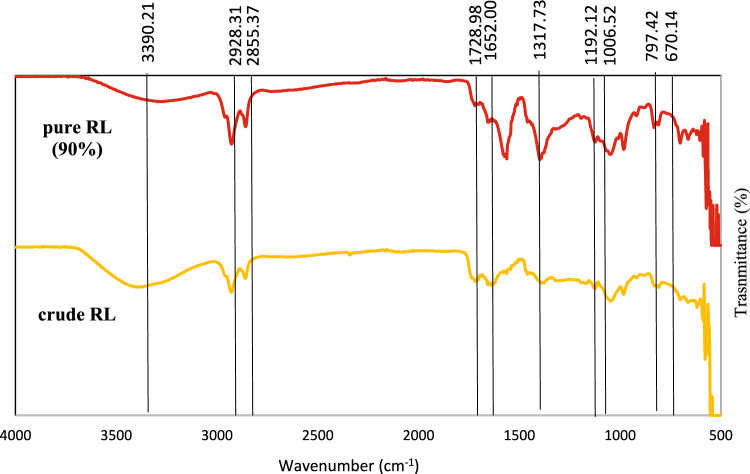
FTIR spectra of pure rhamnolipid; 90% (Agae Technologies, USA) and crude RL produced by *P. aeruginosa* RS6

Figure 3 represents the FT-IR profile of crude RL and standard RL (90% purity). Important absorption spectra of crude RL were observed at 3390.21 cm^−1^, 2928.31 cm^−1^, 2857.37 cm^−1^, and 1730.96 cm ^−1^. The band at 3390.21 cm^−1^ corresponds to -OH stretching of the hydroxyl group. The peaks at 2928.31 cm^−1^ and 2855.37 cm^−1^ represent -CH aliphatic stretching and -CH_2_ methyl carbon stretching, respectively. The peak at 1728.98 cm^−1^ corresponds to the -C = O stretching of carbonyl groups. The -OH stretching of the hydroxyl group, -CH aliphatic stretching, -CH_2_ methyl carbon stretching, and ester carbonyl group (-C = O) affirm the lipid moiety of glycolipids such as rhamnolipids (Ibrahim 2017). This finding is similar to the FTIR spectra of rhamnolipid produced from *P. aeruginosa* UKMP14T reported by Sabturani et al. (2016).

### Antifungal activity of lemongrass EO

Lemongrass EO demonstrated dose-dependent inhibition of mycelial growth in all the tested phytopathogens. At the lowest concentration tested (0.8 mg/mL), the inhibition ranged from 27.39% against *G. boninense* to 44.72% against *F. oxysporum* and 54.6% against *R. microporus*. It showed complete inhibition of mycelial growth at 3.2 mg/mL against *R. microporus* and *F. oxysporum* and at 4 mg/mL against *G. boninense* (Table 2). Increasing the concentration progressively enhanced the antifungal effect. Complete inhibition (100%) of *R. microporus* was achieved at 3.2 mg/mL, whereas *G. boninense* required 4 mg/mL for complete suppression. *F. oxysporum* showed total inhibition at 3.2 mg/mL. The positive control resulted in 100% inhibition for all tested pathogens, while the negative control showed no inhibition. The MFC was determined to be 4.0 mg/mL for *R. microporus* and *G. boninense* and 3.2 mg/mL for *F. oxysporum* (Table 3). These results demonstrate that the lemongrass EO exhibits both fungistatic and fungicidal properties against the evaluated fungal phytopathogens.

**Table 2:** Inhibitory effects of lemongrass (*C. flexuosus*) EO on the mycelial growth of fungal pathogens

Phytopathogen	Percentage inhibition of mycelial growth (%)						
	0.8 mg/mL	1.6 mg/mL	2.4 mg/mL	3.2 mg/mL	4 mg/mL	PC	NC
*Rigidoporus microporus*	54.6 ± 0.8^a^	66.37 ± 0.5^b^	79.1 ± 0.8^c^	100 ± 0^d^	100 ± 0^d^	100 ± 0^d^	0^e^
*Ganoderma boninense*	27.39 ± 0.9^a^	39.43 ± 0.6^b^	54.61 ± 1.0^c^	77.14 ± 0.7^d^	100 ± 0^e^	100 ± 0^e^	0^f^
*Fusarium oxysporum*	44.72 ± 0.6^a^	57.14 ± 0.6^b^	75.4 ± 1.0^c^	100 ± 0^d^	100 ± 0^e^	100 ± 0^e^	0^f^

*PC* positive control (Benomyl 50WP 4 mg/mL), *NC* negative control (sterile distilled water)

The results are expressed as mean (n = 3), mean ± SD; measured after full mycelial growth in the control plate

**Table 3:** MIC and MFC values of lemongrass EO against different phytopathogens

No	Phytopathogen	MIC (mg/mL)	MFC (mg/mL)
1	*Rigidoporus microporus*	3.2	4.0
2	*Ganoderma boninense*	4.0	4.0
3	*Fusarium oxysporum*	3.2	3.2

*MIC* minimum inhibitory concentration, *MFC* minimum fungicidal concentration

The results are expressed as mean (n = 3), mean ± SD; measured after full mycelial growth in the control plate

Zhang et al. (2022) reported a half-maximal inhibitory concentration (IC_50_) value of 0.229 μL/mL against *Fusarium avenaceum*. Additionally, the inhibitory effect against fungal pathogens, *F. oxysporum* f. sp. *cepa* and *Sclerotium oryzae* was recorded with IC_50_ values of 0.739 ± 0.03 µL/plate and 0.420 ± 0.05 µL/plate, respectively (Devi et al. 2021). A study conducted by Da Silva Gundel et al. (2018) on the antifungal efficacy of lemongrass EO against human pathogens, *Candida albicans* and *Cryptococcus neoformans,* reported MIC values of 1.22 mg/mL and 0.58 mg/mL, respectively. Gao et al. (2020) reported MIC values of 0.0781%, 0.039%, and 0.0781% against planktonic C*. albicans, Candida tropicalis,* and *Staphylococcus aureus,* respectively. Lemongrass EO tested against *C. tropicalis* MTCC1000 and *C. parapsilosis* MTCC998 reported MIC values of 1.25 µL/mL and 2.5 µL/mL and MFC values of 2.5 µL/mL and 5.0 µL/mL, respectively (Al-Ghanayem 2023). The antifungal property of lemongrass EO is primarily attributed to its major volatile chemical constituents, which include citral, geranyl acetate, geraniol, and isogeranial (Mwithiga et al. 2022). The antifungal mechanism of lemongrass EO includes the inhibition of fungal growth by disrupting crucial cellular activities such as membrane formation, respiration, and spore germination alongside inhibiting fungal enzymes required for its growth and metabolism (Al-Ghanayem 2022).

### Stability evaluation

Creaming was observed in formulation A1 after four weeks of storage following centrifugation. Creaming in nanoemulsions is linked to the density difference between the oil and water phases and it can increase the likelihood of droplet coalescence by inducing greater interaction between emulsion droplets (Wilson et al. 2021). Formulations A2 and A3 remained monophasic throughout the storage period. At higher surfactant volumes, the steric hindrance provided by the biosurfactant, combined with the effects of sonication, ensured the stability of the nanoemulsions under centrifugal shear (Carpenter and Saharan, 2016). In the thermal stability tests, all the formulations exhibited a single phase at both the tested temperatures (4 °C and 54 °C), except for formulation A1, which phase separated at the tested temperatures. Phase separation in emulsion systems may arise from a significant increase in particle size over time (Prasert and Gohtani 2016).

### Particle size and PDI analysis

**Fig. 4:**
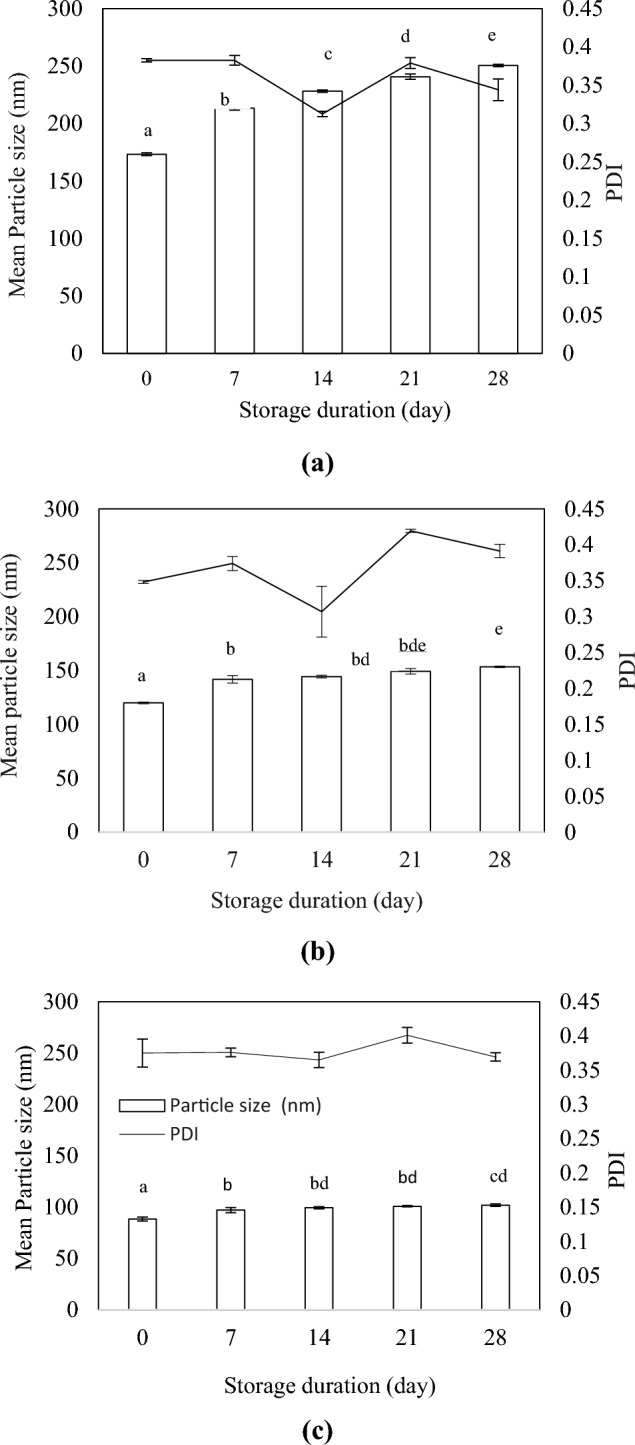
Mean particle size and polydispersity index (PDI) of (**a**) Formulation A1, (**b**) Formulation A2 and (**c**) Formulation A3 at day 0, 7, 14, 21 and 28

The mean particle size and PDI of the developed nanoemulsions were evaluated for 28 days (Fig. 4). The mean particle sizes of formulations A1, A2, and A3 were 173.35 nm, 119.95 nm, and 88.4 nm, respectively, on day 1. The mean particle size decreased with increasing surfactant volume. A slight increase in the mean particle size was observed in all the formulations over the weeks, however, the mean particle size of formulation A2 (153.47 nm) and formulation A3 (101.93 nm) remained within the optimal range (< 200 nm) after 28 days of storage at room temperature. The mean particle size of formulation A1 increased significantly to 250.5 nm ± 0.99 on day 28. The steep increase in the mean particle size was due to droplet growth via Ostwald ripening during storage (Wooster et al. 2016). These results indicate that RL-stabilized nanoemulsions are relatively stable to storage, provided an adequate amount of biosurfactant is used to emulsify the oil droplets. The PDI values of formulations A1, A2, and A3 remained below 0.5 until day 28, which indicate the monomodality of the nanoemulsions. Bai and McClements (2016) developed a medium-chain triglyceride (MCT) oil nanoemulsion stabilized by RL (0.2%) through microfluidization (13 kpsi, three cycles) and reported a droplet size of 110 nm. Onaizi (2021) reported that the mean droplet sizes of ultrasonically produced emulsions (oil-to-water ratio: 1:4) reduced from 178 to 53 nm when stabilized using 0.5% and 4% of RL, respectively. Li et al. (2018) reported a similar pattern in the reduction of particle size of concentrated emulsions, whereby the droplet size reduced from ~ 800 nm to ~ 310 nm when emulsified by 0.5% and 3% RL, respectively. Additionally, Al-Sakkaf and Onaizi (2023a, b) developed a crude oil nanoemulsion through ultrasonication (15 min; 30/3 s on/off pulse) employing 2% RL and reported a droplet size of 102 nm.

Emulsifiers play a key role in reducing the oil–water interfacial tension, thereby stabilizing the emulsion systems. Appropriate type, concentration, and volume of emulsifier are crucial in ensuring the long-term stability of the developed nanoemulsions (Mushtaq et al. 2023). Besides the role of emulsifiers, the magnitude of mechanical force applied to the emulsions critically influences droplet disruption. In this study, sonication process was used to develop the nanoemulsions. Ultrasound homogenization is used to develop nanoemulsions with small particle size and long-term stability. The ultrasonic waves released by the sonicator probe produce cavitation and shear force, leading to the formation of cavitation bubbles. The subsequent collapse of these bubbles produces intense turbulence, which promotes droplet deformation and size reduction. (Shah et al. 2024).

### pH and zeta potential assessment

**Fig. 5:**
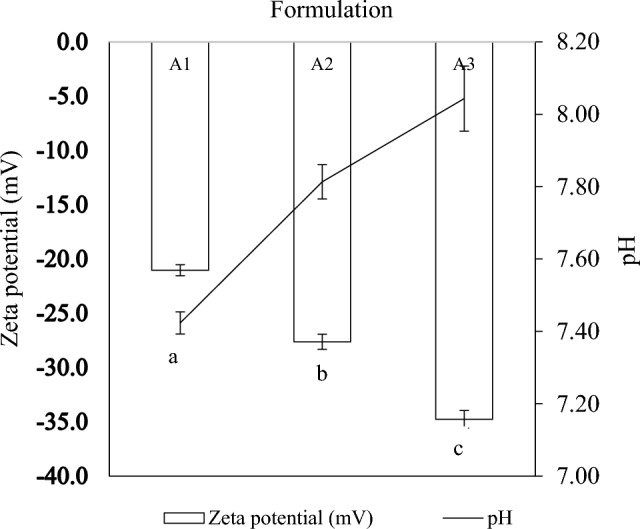
Zeta potential as a function of pH of formulations A1, A2, and A3

The zeta potential of the RL-stabilized nanoemulsions was measured as a function of pH (Fig. 5). Zeta potential reflects the surface charge density of developed nanoemulsions (Preeti et al. 2023), and pH is one of the essential factors that affect the zeta potential values. The zeta potential increased from − 21.04 mV at pH 7.42 in formulation A1 to − 27.63 mV at pH 7.81 ± 0.05 in formulation A2 and − 34.77 mV at pH 8.04 in formulation A3. The zeta potential values increased with rising pH. Sufficient surfactant molecules are required to adsorb to the oil–water interface and form a stable interfacial film. This ensures good colloidal stability of nanoemulsions. For nanoemulsion systems, zeta potential values above − 30 mV signify optimum repulsive force to maintain colloidal stability (Mosa et al. 2023). Nanoemulsions with smaller particle sizes tend to have larger surface charge densities, resulting in greater negative zeta potential values and consequently enhanced stability (Mosa et al. 2023). The good colloidal stability of the RL-coated emulsion droplets can be attributed to the strong electrostatic and repulsive forces between the charged droplets.

The negative charge of the zeta potential values was due to the presence of carboxylic acid groups within the RL molecules. At a high pH, the carboxylic acid group tends to become negatively charged (COO), which induces a rise in the magnitude of negative droplet charge (Bai and McClements 2016). An increase in the emulsion basicity results in higher electrophoretic mobility of the formulation (Kaci et al. 2013). This can be attributed to the deprotonation of carboxylic acid groups in RL molecules at values above the RL pKa (4.28–5.5) (Li et al. 2018). Increased deprotonation of biosurfactant molecules causes a rise in the negative charge on the biomolecules and leads to a higher charge density on the droplet surface of the nanoemulsions (Kumar and Mahto 2017).

### Viscosity determination

**Fig. 6:**
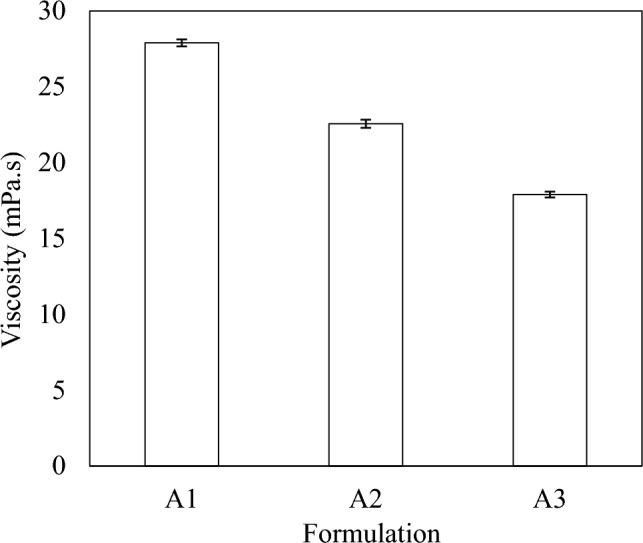
Viscosity of Formulation A1, A2 and A3

The viscosities of formulations A1, A2, and A3 were 27.89 mPa/s, 22.56 mPa/s, and 17.9 mPa/s, respectively (Fig. 6). The viscosity values gradually decreased with increasing concentration of RL. Viscosity assessment is crucial to determine the type of emulsions developed. Emulsion systems with low viscosity depict an O/W solution, while high viscosity affirms a water-in-oil (W/O) solution (Gurpreet and Singh 2018). For pesticide formulations, an O/W emulsion system is more favourable (Feng et al. 2018). The processing parameters of sonication can be optimized to reduce nanoemulsion viscosity. For instance, increasing sonication time reduces nanoemulsion viscosity and enhances stability (Mosa et al. 2023). Factors that influence the viscosity of emulsions include oil and emulsifier concentration, shear rate, and temperature (Sun et al. 2025). Al-Sakkaf and Onaizi (2022) reported that the viscosity of crude oil nanoemulsion stabilized by RL was below 7 mPa/s even at high RL concentration (4 wt.%). The difference in the value of viscosity with our findings might be due to the difference in the droplet size of the formulations. A mean droplet size of 42.4 nm was reported in that study, which is much lower than the value reported in the current study (88.4 nm–173.35 nm).

### Optimization of nanoemulsion

**Fig. 7:**
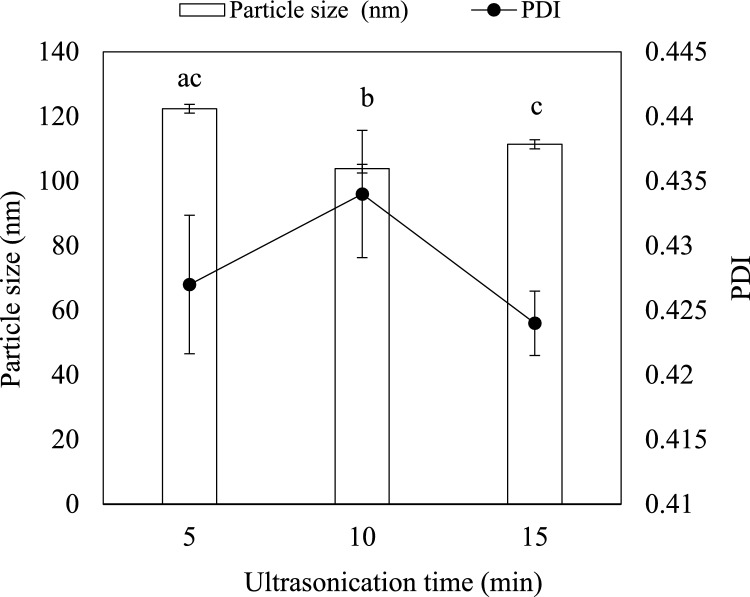
The mean particle size and PDI of optimized RL – stabilized nanoemulsion at different sonication time

Formulation A2 was selected for optimization of sonication time to achieve a stable nanoemulsion with desirable physicochemical properties. The effect of sonication time on droplet size and PDI was investigated by subjecting the formulation to ultrasonication for 5, 10, and 15 min (Fig. 7). The mean particle size decreased from 122.4 nm at 5 min to 103.9 nm at 10 min, followed by a gradual increase to 111.4 nm at 15 min. The mechanical energy released through cavitation during sonication assists droplet fragmentation, leading to a reduction in particle size (Wang et al. 2022). Qamar et al. (2019) reported that longer sonication time reduces the particle size of nanoemulsions. A similar pattern of data was reported by Zou et al. (2020).

However, prolonged sonication may lead to droplet coalescence due to excess surfactant molecules that fail to adsorb completely at the oil–water interface (Ettoumi et al. 2016). This incidence may also be attributed to the “overprocessing” of the emulsion (Kentish et al. 2008). The PDI value at 10 min of sonication (0.434) was slightly higher than the PDI values reported at 5 min (0.427) and 15 min (0.424) of sonication. However, the PDI values at all the three sonication durations remained below 0.5, indicating a monomodal distribution of droplets. Hence, 10 min of sonication was determined to be the optimum sonication time, as it yielded both a low droplet size and a low PDI.

## Conclusion

Lemongrass EO exhibited antifungal effects at low concentrations of 0.8–4 mg/mL, effectively suppressing the growth of plant pathogens *G. boninense, R. microporus*, and *F*. *oxysporum*. RLs exhibited good emulsifying activity and reported higher surface tension reduction capacity than SDS. To overcome issues of volatilization and degradation of EO compounds, the encapsulation of EO into nanoemulsion systems was carried out using the ultrasonication method. Formulation A2 exhibited a low mean particle size and PDI, along with a high absolute zeta potential, and low surface tension. The nanoformulation remained stable during 28 days of storage at ambient temperature. These findings demonstrate that the production of a bio-based nanoemulsion, stabilized by RL possesses good physicochemical and functional stability, providing avenues to broaden the application of RL-stabilized nanoemulsions in various sectors.

## Data Availability

Detailed data are available in the manuscript.
